# Association of Mouse Mammary Tumor Virus With Human Breast Cancer: Histology, Immunohistochemistry and Polymerase Chain Reaction Analyses

**DOI:** 10.3389/fonc.2018.00141

**Published:** 2018-05-07

**Authors:** James S. Lawson, Chiara Mazzanti, Prospero Civita, Michele Menicagli, Christopher C. Ngan, Noel J. Whitaker, Jacob Hochman, Ori Braitbard, Benafsha Yosufi, Wendy K. Glenn

**Affiliations:** ^1^University of New South Wales, Sydney, NSW, Australia; ^2^Fondazione Pisana per la Scienza Onlus, Pisa, Italy; ^3^Department of Cell and Developmental Biology, Alexander Silberman Institute of Life Sciences, The Hebrew University of Jerusalem, Jerusalem, Israel; ^4^Douglass Hanly Moir Pathology, Macquarie Park, NSW, Australia

**Keywords:** breast cancer, mouse mammary tumors, mouse mammary tumor virus, histology, mouse mammary tumor virus signal peptide, mouse mammary tumor virus p14, immunohistochemistry, polymerase chain reaction

## Abstract

**Purpose:**

The purpose of this study is to determine whether mouse mammary tumor virus (MMTV)-associated human breast cancer has the same or similar histology to MMTV-associated mouse mammary tumors. Such associations may indicate a role for MMTV in human breast cancer.

**Methods:**

Immunohistochemical techniques (using antibodies directed against the signal peptide p14 of the envelope precursor protein of MMTV) and polymerase chain reaction (PCR) analyses were used to identify MMTV proteins and MMTV-like envelope gene sequences in a series of breast cancers from Australian women. The histological characteristics of these human breast cancer specimens were compared with MMTV positive mouse mammary tumors. The same methods were used to study benign breast tissues which 1–11 years later developed into breast cancer.

**Results:**

MMTV p14 proteins were identified in 27 (54%) of 50 human breast cancers. MMTV *env* gene sequences were identified by PCR in 12 (27%) of 45 human breast cancers. There was a significant correlation between the presence of MMTV (identified by p14 immunohistochemistry) in human breast cancers and histological characteristics similar to MMTV positive mouse mammary tumors (*p* = 0.001). There was a non-significant correlation between the presence of MMTV *env* gene sequences (identified by PCR) in human breast cancers and histological characteristics similar to MMTV positive mouse mammary tumors (*p* = 0.290). MMTV p14 proteins were identified in 7 (54%) of 13 benign breast specimens that later developed into human breast cancers. MMTV by PCR was identified in two benign specimens one of whom later developed MMTV positive breast cancer.

**Discussion:**

These observations offer evidence that MMTV may be associated with characteristic human breast cancer histology. p14-based immunohistochemistry appears to be a more reliable technique than PCR for the identification of MMTV in human breast cancer. Identification of MMTV-associated p14 proteins in benign breast tissues confirms prior PCR-based studies that MMTV infection occurs before the development of MMTV positive breast cancer.

**Conclusion:**

Many MMTV positive human breast cancers have similar histology to MMTV positive mouse mammary tumors. MMTV infection identified in benign breast tissues precedes development of MMTV positive human breast cancer. When considered in the context of prior studies, these observations indicate a likely role for MMTV in human breast cancer.

## Purpose

The purpose of this study is to determine whether mouse mammary tumor virus (MMTV)-associated human breast cancer has the same or similar histology to MMTV-associated mouse mammary tumors. Such associations may indicate a role for MMTV in human breast cancer. MMTVs have a documented causal role in mouse mammary tumors (1). Viral sequences, virtually identical to MMTV, have been repeatedly identified in human breast cancers by polymerase chain reaction (PCR) laboratory techniques and more recently by massive parallel sequencing (2, 3). The evidence suggestive of a role for an MMTV-like virus in human breast cancer is substantial but not conclusive.

## Context of This Study

It is helpful to place this current study in the context of past investigations. A “mouse milk factor,” later identified as MMTV, was first associated with mouse mammary tumors by Bittner in 1936 (4). MMTV exerts oncogenic influences on both wild (feral) and inbred laboratory mice which leads to consistent histology patterns in mouse mammary tumors (5). During the 1950s, Thelma Dunn studied the histological characteristics of mammary tumors in wild and laboratory mice. Over 90% of mammary tumors in feral mice are adenocarcinomas. Dunn classified mouse mammary tumors as types A and B. Type B is by far the most common type. There are additional types of mouse mammary tumors including papillomas but these are uncommon.

The first observation that some human breast cancers had similar histology to MMTV-associated mouse mammary tumors was made by Wellings (6). However, others disagree that there are such similarities (7). The view that human breast cancer histology differs from mouse mammary tumors may be based on observations of advanced breast cancers. Many, but not all, advanced breast cancers are characterized by streams of elongated cancer cells surrounded by dense connective tissues. By way of contrast, the observations by Wellings appear to be based on the early proliferative stages of both human breast neoplasia and mouse mammary tumors (6). In our opinion, it is valid to compare these early stages of human breast cancer with mouse mammary tumors.

## Comparisons Between MMTV-Associated Mouse Mammary Tumors and MMTV-Associated Human Breast Cancer

There are close parallels between MMTV-associated cancer biology in both human breast cancer and mouse mammary tumors. (i) MMTV has been identified globally in human breast cancer and mouse mammary tumors (2, 8), (ii) MMTV virus particles have been identified in human breast cancers and mouse mammary tumors (9, 10), (iii) MMTV has been identified in both mouse and human milk (1, 11, 12), (iv) nucleotide sequences and structure of the 9,900-bp long MMTV genome is 84–98% homologous in both mouse and human breast cancer (3, 13–16), (v) abnormal cancer related gene expression is similar (17, 18), (vi) MMTV-associated protein expression is similar (19–21), (vii) MMTV superantigen expression required for activating lymphocytes appears to be the same (22–24), (viii) MMTV infects intestinal B lymphocytes and randomly integrates into both the mouse and human genome (1, 25–27), (ix) hormone responsiveness appears to be similar (28–30), (x) MMTV-associated breast cancer histology may be similar (5, 6, 31), (xi) there is a positive serological response to MMTV in both mouse and humans although this has not been confirmed by modern methods in humans (32–36), and (xii) MMTV is present in normal human breast and mouse mammary tissues prior to the development of MMTV positive breast cancer and mouse mammary tumors (37–39).

## MMTV p14 Protein

p14 is the signal peptide of the envelope precursor protein of MMTV (21). It has been developed by the Hochman group in Israel as an antibody which can be used for immunohistochemistry analyses to identify MMTV. In addition to being a signal peptide, it is a multifunctional 98 amino acid peptide. It binds and transcriptionally regulates key proteins of the cellular stress response and ribosome biogenesis. p14 can also function in both an oncogenic and an anti-oncogenic capacity depending on its phosphorylation status (40). Furthermore, p14 is expressed on the cell surface of murine mammary carcinomas and lymphomas that harbor MMTV (40). p14 has recently been located on the cell surface of primary human breast cancer cells grown in culture (41). The same human breast cancer cells were also positive for MMTV *env* sequences using PCR. p14 has been used for vaccination and passive immunization (using monoclonal antibodies and adoptive cell transfer) in MMTV positive mouse mammary tumors (41).

The use of MMTV p14 antibodies in immunohistochemistry analyses, offers improved consistency in the identification of MMTV in human breast cancer as compared to PCR techniques (21, 40, 41).

## Hypothesis

We hypothesize that because of this almost identical cancer-associated biology between MMTV positive human breast cancer and mouse mammary tumors^1^, the cancer histology should also be similar. We have previously demonstrated that this hypothesis may be correct (31). However, in the previous study, correlations between human and mouse histology and the identification of MMTV, were not statistically significant (31). These insignificant results were probably due to the inconsistent identification of MMTV by PCR. Advances in identifying MMTV by immunohistochemistry using p14 antibody has added validity to the hypothesis.

## Materials and Methods

### Ethics

This project was formally considered and approved by the Human Research Ethics Committee of the University of New South Wales (UNSW), Sydney, Australia. Ref: HC11421.

### Materials

Fifty-five breast cancer specimens from Australian women were included in this study. All the specimens were from the archives of an Australian pathology service (Douglass Hanly Moir—Pathology) and had been formalin fixed and paraffin mounted. A study based on PCR analyses of MMTV in these specimens has previously been published (39). Additional specimens were invasive breast cancers from women who had previously had benign breast conditions and who several years later developed breast cancer. These specimens all differ from those used in our previous publications (31, 42).

Ten archival MMTV positive mouse mammary tumors in C3H mice were from the Jackson laboratories (ME, USA).

### Investigations Based on PCR

The DNA extraction and detection of MMTV-like *env* sequences were performed by PCR techniques as described by Wang et al. (43). These PCR analyses were conducted in three independent laboratories—the Icahn School of Medicine at Mount Sinai (ISMMS) (New York), the UNSW, Sydney, Australia, and the Fondazione Pisana per la Scienza Onlus, Pisa, Italy. The results of these PCR analyses have previously been published (39). The primer sequences used in these PCR analyses include part of the MMTV *env* gene, which differs from human endogenous retrovirus 10.

Contamination is a well-known problem with PCR analyses. Therefore, with respect to specimens analyzed at ISMMS, the reagents and PCR products were tested for the presence of murine mitochondrial (MoMt) and genomic DNA. The methods used were as described by Deligdisch et al. (44).

The same PCR methods were used to identify MMTVs in the 10 archival mouse mammary tumors. The results have previously been reported (45).

### Investigations Based on Immunohistochemistry

p14 antibodies used in this current study were prepared as previously described using purified recombinant protein (21). This protein, designated p14 (based on Western blotting analyses), corresponds to the 98 amino acids of the MMTV envelope precursor signal peptide (46). Antibodies to p14 appear to be specific for the identification of MMTV (21, 40, 41, 46).

The immunohistochemical analyses were conducted on the same specimens in both the UNSW and the Fondazione Pisana per la Scienza Onlus, Pisa, Italy. The same methods were used in each independent laboratory. The immunohistochemical analyses were performed on 5 μm-thick paraffin sections. The antigen retrieval was achieved with MS-unmasker solution (DIAPATH, Martinengo, BG, Italy) in microwave. Histostain–Plus kit (Invitrogen, Carlsbad, CA, USA) was used according to manufacturer’s protocol. The slides were incubated for 2 h with a primary antibody, rabbit polyclonal anti-MMTV-p14 (1:500 dilution), then developed with diaminobenzidine chromogen (DAB) (DAKO, Glostrup, Denmark), and counterstained with hematoxylin. Negative controls included the omission of the primary antibody.

The results of immunohistochemical analyses for estrogen receptor (ER), progesterone receptor (PR), human epidermal growth factor receptor 2 (HER2), and p53 were available for each specimen as part of standard laboratory procedures at Douglass Hanly Moir Pathology—the source of the specimens. The grade of invasive breast cancers was available for 20 of the specimens.

### Immunohistochemistry Identification of Lymphocytes

In human tissue specimens, lymphocytes can be readily identified by their histological characteristics, mainly because of their smaller diameter when compared to human breast cancer cells. In mouse tissue specimens, the diameter of mouse mammary tumor cells is approximately 20% less than human breast cancer cells. Therefore, when making comparisons of the histological characteristics between human and mouse mammary tumor cells, it is important to exclude lymphocytes. Lymphocytes plus normal and cancerous epithelial cells in human specimens can be identified by specific antibodies. In this study, CD45 antibodies (Dako M0701), which are specific for the identification of both T and B lymphocytes, were identified by automated immunochemistry techniques at the Douglass Hanly Moir—Pathology laboratories.

### Histological Comparisons Between Human Breast Cancer and Mouse Mammary Tumors

The histological characteristics of both human breast cancer and mouse mammary tumor specimens were independently assessed by authors of this current study—James S. Lawson and Wendy K. Glenn. These specimens were sectioned and stained with hematoxylin and eosin (H&E). The histological assessment was conducted “blind,” that is, the histological assessments were conducted without knowledge of the outcomes of the PCR or immunohistochemistry p14 analyses. When viewed macroscopically human breast cancer and mouse mammary tumors have different characteristics. When examined microscopically at a magnification of 100, 200, and 400, some human breast cancers and mouse mammary tumor tissues are very similar (31).

Comparisons were also made between the histology of 12 human benign breast specimens which 1–11 years later developed into breast cancer.

### Statistics

The Pearson’s correlation test was used to determine if there were correlations between the identification of MMTV by immunohistochemistry (using p14 antibodies) and PCR and (i) specific histological features in human breast cancer and (ii) biomarkers including ERs, PRs, HER2, and p53. A *p*-value of less than 0.05 was considered to be significant. The SPSS statistical package was used.

## Results

### Comparisons of Histology Between MMTV Positive Human Breast Cancer and MMTV Positive Mouse Mammary Tumors

Mouse mammary tumor virus p14 proteins were identified in 27 (54%) of 50 human breast cancers. MMTV *env* gene sequences were identified by PCR in 12 (27%) of 45 human breast cancers. There was a significant correlation between the presence of MMTV (determined by immunohistochemistry using p14 antibodies) in human breast cancers and histological characteristics similar to MMTV positive mouse mammary tumors (*p* = 0.033) based on UNSW laboratory data, *p* = 0.001 using the combined data from Pisa and UNSW (Table S1 in Supplementary Material).

There was no correlation between the presence of MMTV *env* gene sequences identified by PCR in human breast cancers and histological characteristics similar to MMTV positive mouse mammary tumors (*p* = 0.290). There was no correlation between the identification of MMTV by immunohistochemistry using p14 antibodies and PCR (*p* = 0.932).

These results are shown in Tables 1 and 2 and Tables S1–S3 in Supplementary Material. There are gaps in the data shown in Table S2 in Supplementary Material, these are due to the absence of beta-globin or exhaustion of the specimen blocks. There were no differences between invasive and non-invasive breast neoplasia with respect to their similarity or non-similarity with mouse mammary tumor histology (Table S2 in Supplementary Material).

**Table 1:** Human breast cancer.

	Histology Dunn positive	Histology Dunn negative
MMTV by p14 positive	19	8
MMTV by p14 negative	6	17

*Correlation between identification of MMTV by p14 immunohistochemistry (Pisa and University of New South Wales data combined) and histological characteristics. Breast cancer histology assessed as similar or not similar to MMTV positive mouse mammary tumors Dunn types A and B. There is a significant correlation between the presence or absence of MMTV by immunohistochemistry using p14 antibodies and histological characteristics (*p* = 0.001 significant)*.

*MMTV, mouse mammary tumor virus*.

**Table 2:** Human breast cancer.

	Histology Dunn positive	Histology Dunn negative
MMTV by PCR positive	8	4
MMTV by PCR negative	16	17

*Correlation between identification of MMTV by PCR and histological characteristics. Breast cancer histology assessed as similar or not similar to MMTV positive mouse mammary tumors Dunn types A and B. There is no correlation between the presence or absence of MMTV identified by PCR and histological characteristics (*p* = 0.290 not significant)*.

*MMTV, mouse mammary tumor virus; PCR, polymerase chain reaction*.

These observations offer evidence that MMTV may be associated with characteristic human breast cancer histology.

Dunn type A MMTV positive human breast cancers have intensely stained nuclei with intact alveolar milk acini (31). Dunn type B MMTV positive human breast cancers are also characterized by intensely stained nuclei which occupy most of the cell (31). These cells are round and regular in size and are clumped together without glandular acini or lumina (31).

**Figure 1:**
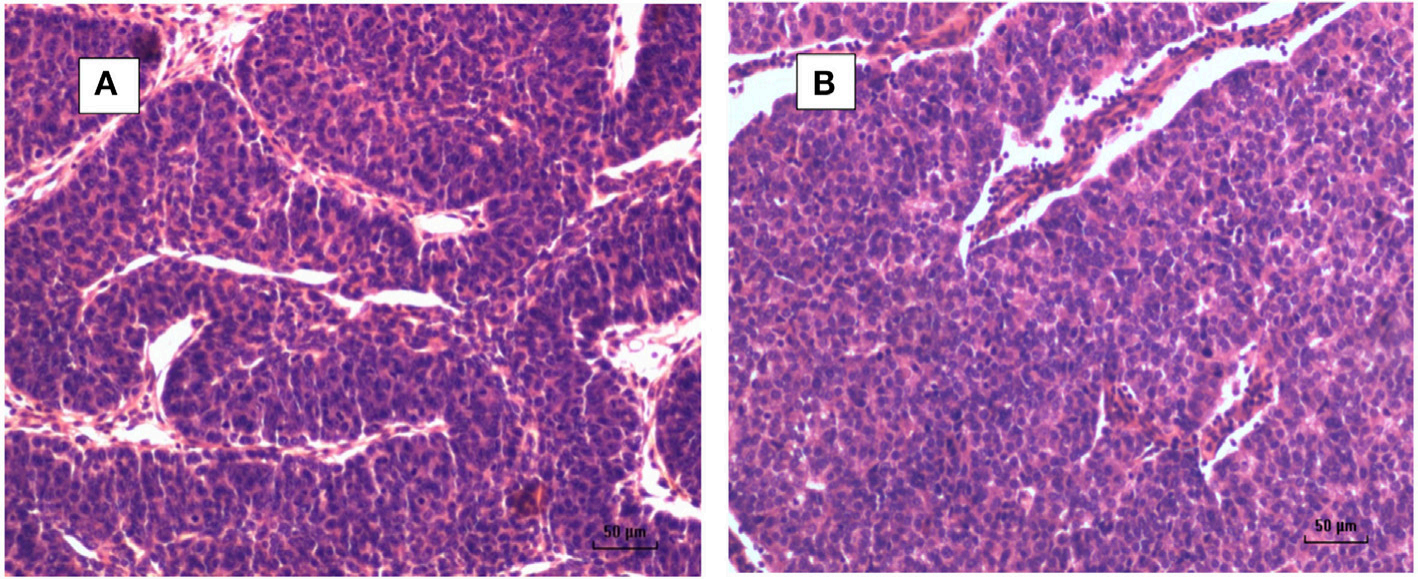
**(A)** Mouse mammary tumor—Dunn type B, mouse mammary tumor virus (MMTV) positive by polymerase chain reaction (PCR). **(B)** Human invasive ductal carcinoma—similar histology to Dunn type B mouse mammary tumor. MMTV positive by PCR.

**Figure 2:**
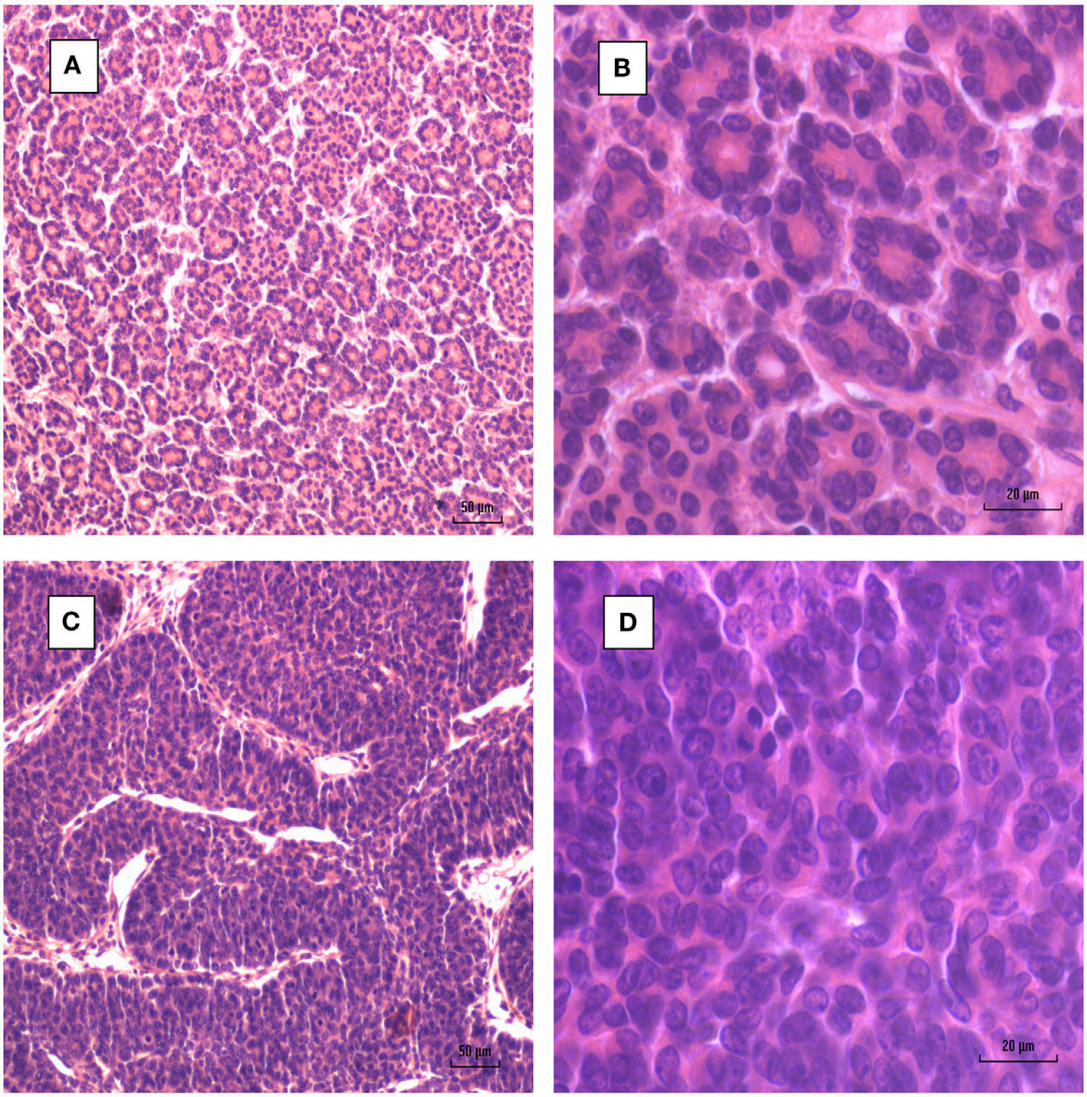
Mouse mammary tumors. **(A,B)** Dunn type A. **(C,D)** Dunn type B. Dunn type B is much more prevalent than Dunn type A.

**Figure 3:**
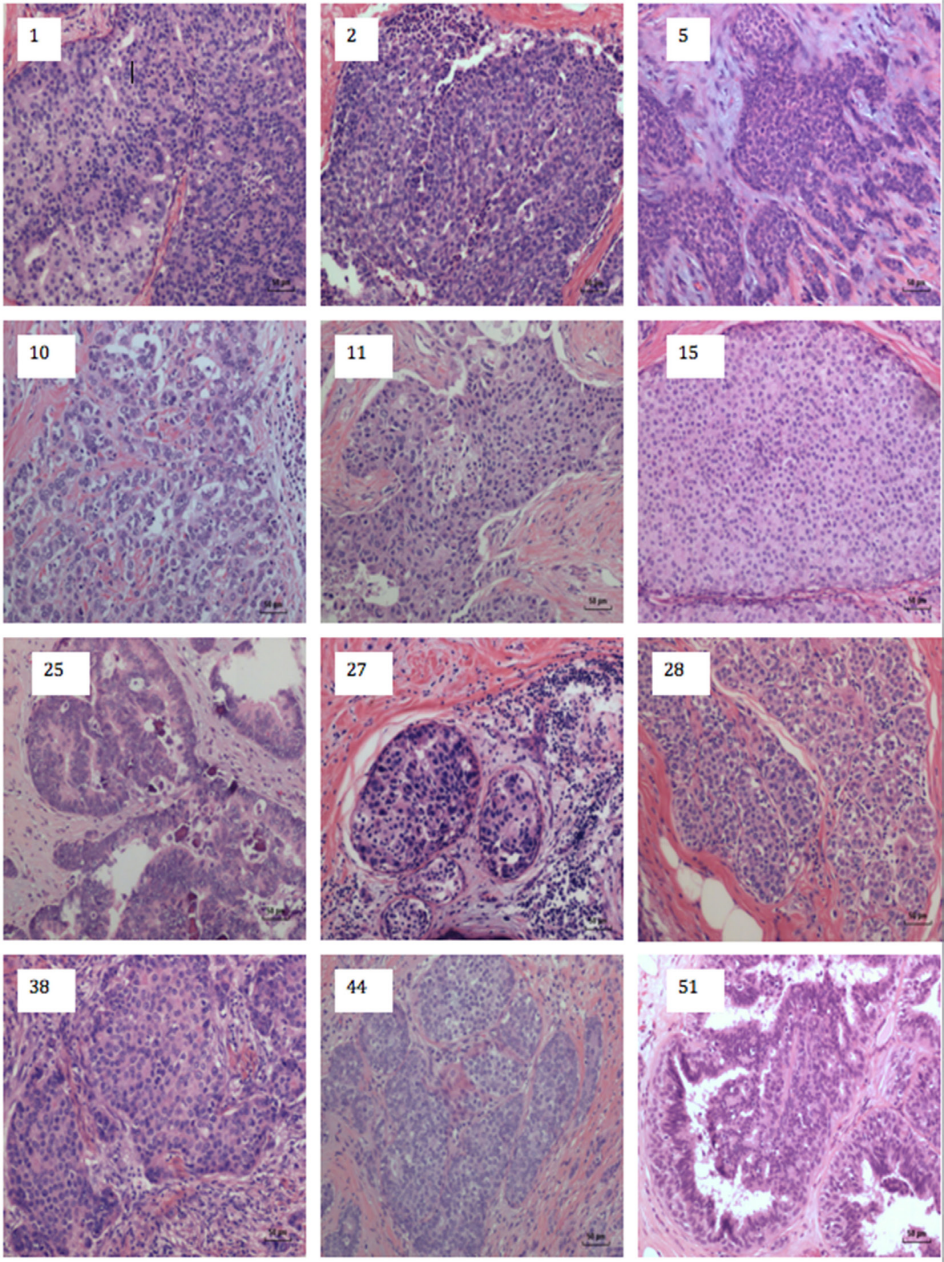
Human breast cancer specimens with histological characteristics similar to mouse mammary tumors. The numbers on each image refers to the patient numbers listed in Table S1 in Supplementary Material.

A typical MMTV positive human breast cancer specimen with Dunn type B histological characteristics compared to MMTV positive Dunn type B mouse mammary tumor is shown in Figures 1 and 2. A series of human breast cancers with histological characteristics similar to mouse Dunn type B are shown in Figure 3. The numbers on each image corresponds to patients listed in Table S2 in Supplementary Material. Typical Dunn type B human breast cancers are much more common than Dunn type A.

**Figure 4:**
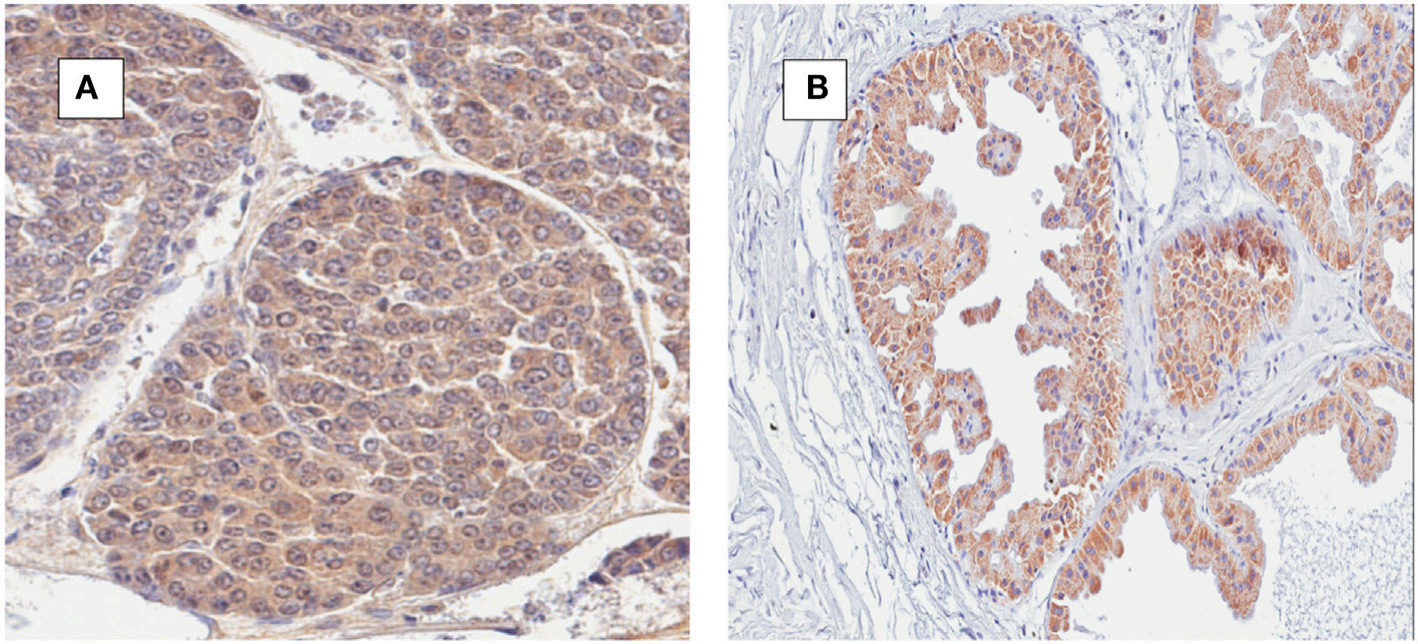
**(A)** Mouse mammary tumor; mouse mammary tumor virus (MMTV) p14 protein positive. **(B)** Human ductal carcinoma *in situ*; MMTV p14 protein positive.

The identification of MMTV by immunohistochemistry using p14 antibodies in both human breast cancer and mouse mammary tumors is shown in Figure 4. There is staining in the cell membranes, cytoplasm, and in some but not all nuclei.

### Infiltrating Lymphocytes

**Figure 5:**
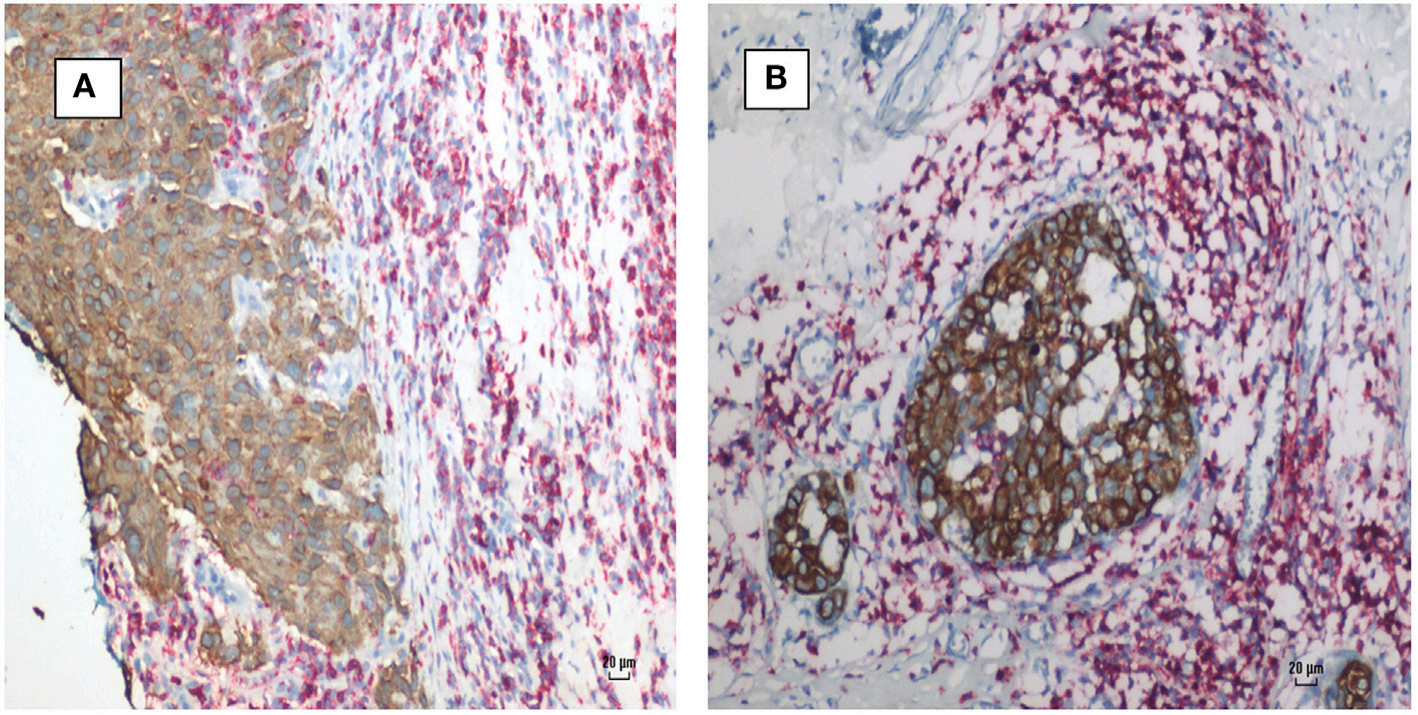
Mouse mammary tumor virus positive human breast cancer. Breast cancer cells (brown stain) and lymphocytes (red stain) by immunohistochemistry using CD45 antibodies. **(A)** Invasive ductal carcinoma. **(B)** Ductal carcinoma *in situ*. Lymphocytes surround and infiltrate both cancers.

Infiltrating lymphocytes were identified in many, but not all, human breast cancer specimens. These lymphocytes could be clearly identified by their histological characteristics and red staining-based immunohistochemistry using antibodies to B and T cells (Figure 5).

### Comparisons Between Human Benign Breast Specimens Which 1–11 Years Later Developed Into Breast Cancer

**Figure 6:**
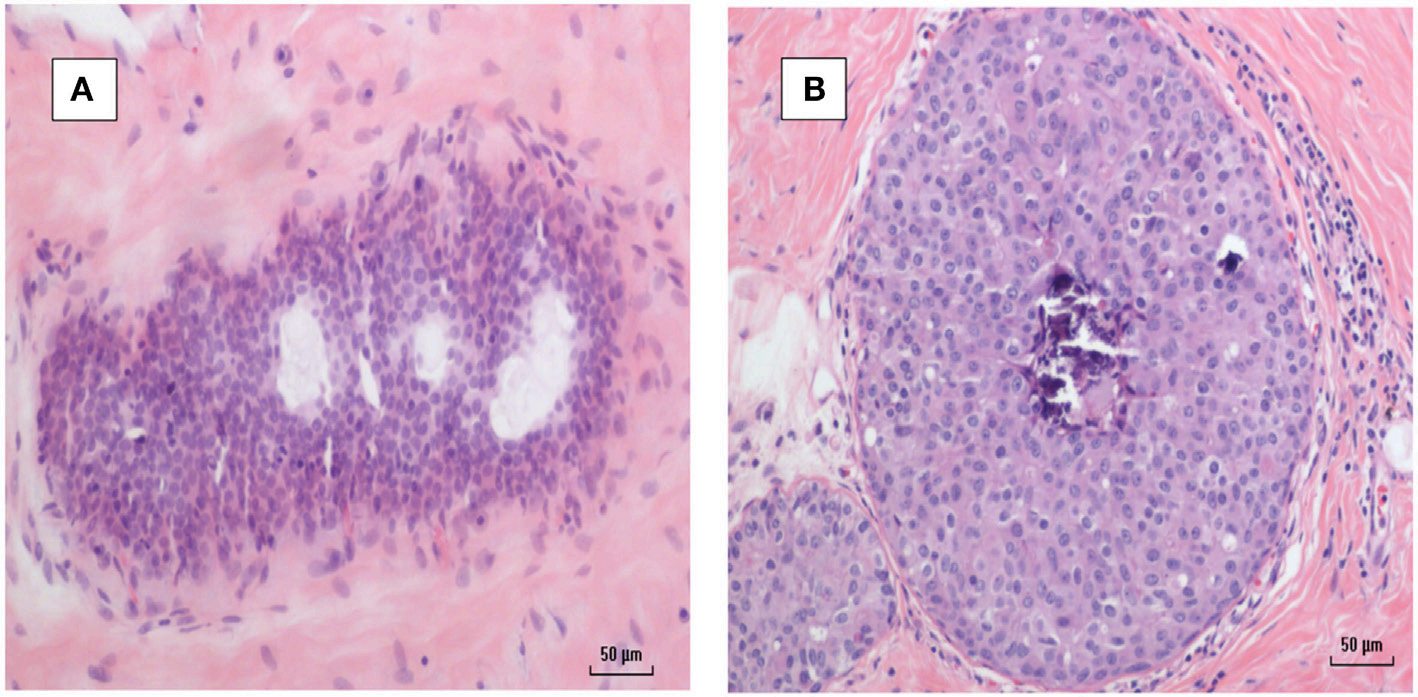
**(A)** Mouse mammary tumor virus (MMTV) positive by polymerase chain reaction (PCR) breast hyperplasia **(B)** MMTV positive by PCR subsequent ductal carcinoma *in situ* breast cancer—same patient.

There were 13 sets of benign breast specimens which 1–11 years later developed into breast cancer. 11 of these 13 benign breast specimens had some hyperplasia (as defined by four or more levels of epithelial cells). MMTV-p14 was identified in 8 of the 13 benign breast specimens which later developed into breast cancer in the same women. Six of the later breast cancers were MMTV-p14 positive. MMTV-p14 was identified in 8 of the 11 benign breast specimens with hyperplasia, 7 of which subsequently developed MMTV-p14 positive breast cancer. The details are shown in Table S3 in Supplementary Material. An MMTV positive benign breast specimen with hyperplasia progressing to MMTV positive breast invasive ductal carcinoma is shown in Figure 6.

### Consistency and Specificity of Immunohistochemistry p14 Analyses Between Laboratories

As shown in Table S2 in Supplementary Material, the identification of MMTV by immunohistochemistry using p14 antibodies is higher than identification by PCR. The identification of MMTV by immunohistochemistry in the Pisa laboratory as compared to the Sydney laboratory (same specimens, same p14 antibody) was the same for 29 specimens and different for 6 specimens.

**Figure 7:**
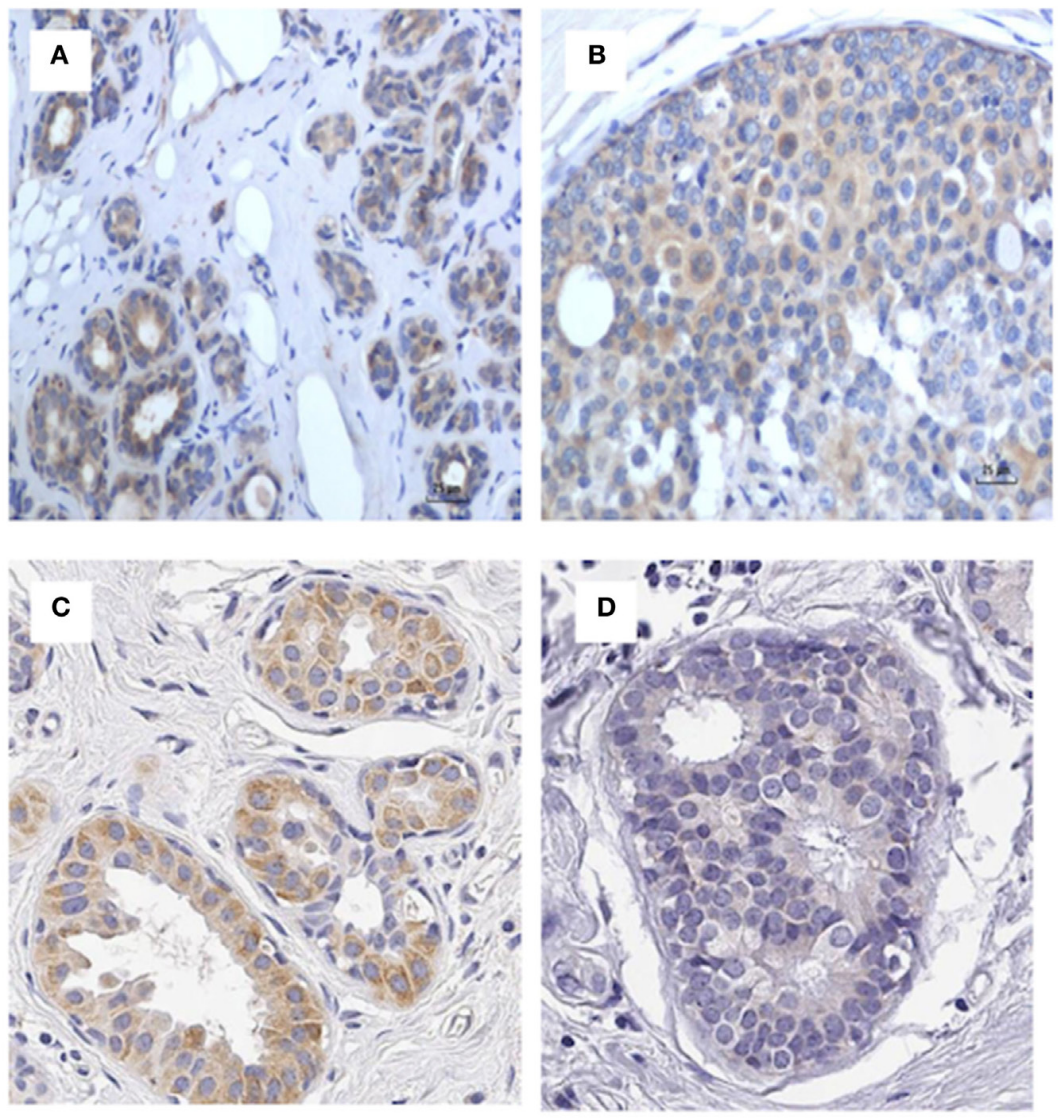
p14 identification in patients 5 and 6 in Table S3 in Supplementary Material indicating specificity of the P14 antibody. **(A)** Patient 5 benign (positive), **(B)** patient 5 cancer (positive), **(C)** patient 6 benign (positive), **(D)** patient 6 cancer (negative). If cross reactivity of the p14 was occurring, the cancer specimen from patient 6 should not be negative.

As shown in Figure 7, identification of MMTV by immunohistochemistry using p14 antibodies in patients 5 and 6 listed in Table S2 in Supplementary Material indicates specificity of the p14 antibody. If cross reactivity of the p14 was occurring, the cancer specimen from patient 6 should not be negative.

### Correlations Between MMTV Identification in Human Breast Cancer, Cancer Grade, and Biomarkers

The results of immunohistochemical analyses for ER, PR, HER2, and p53 are shown in Table S1 in Supplementary Material. There were no significant correlations with respect these biomarkers and MMTV identification in breast cancer by either immunohistochemistry or PCR.

There were no correlations between the breast cancer grade and MMTV identification by either p14 immunohistochemistry or PCR (*p* = 0.586 and 0.719, respectively).

## Discussion

Mouse mammary tumor virus p14 proteins were identified in 25 (50%) of 50 human breast cancers. MMTV *env* gene sequences were identified by PCR in 12 (27%) of 45 of the same human breast cancers. There was a significant correlation between the presence of MMTV (determined by immunohistochemistry using p14 antibodies) in human breast cancers and histological characteristics similar to MMTV positive mouse mammary tumors. This correlation was confirmed in two laboratories (*p* = 0.033 UNSW, *p* = 0.001 Pisa). There was no correlation between the presence of MMTV *env* gene sequences (determined by PCR) in human breast cancers and histological characteristics similar to MMTV positive mouse mammary tumors (*p* = 0.290). There was no correlation between PCR and p14 used for the identification of MMTV (*p* = 0.932).

Identification of MMTV-associated p14 proteins in benign breast tissues confirms prior PCR-based studies that MMTV infection occurs before the development of MMTV positive breast cancer.

These observations offer evidence (i) that MMTV may be associated with characteristic human breast cancer histology and (ii) that MMTV infection precedes the development of MMTV-associated human breast cancer.

There are qualifications with respect to this evidence: (i) due to extremely low levels of viral DNA, the identification of MMTV by PCR in human breast cancers is not consistent, (ii) the assessment of histological characteristics is subjective, and (iii) the assessment of staining used in immunohistochemistry is also subjective (but no less subjective than other analyses based on immunohistochemistry, such as ER and PR protein expression in breast cancer).

### Validity of the Observations

As the p14-based assessments were conducted in two independent laboratories with similar outcomes, the results have added validity.

The identification of extremely low concentrations of retroviral DNA such as MMTV in human breast cancer by PCR is difficult and the results are inconsistent. The results of PCR analyses for the identification of MMTV using the same breast cancer specimens can differ between laboratories (39). There are several reasons for the inconsistent outcomes when PCR is used for the identification of MMTV in human breast cancers: (i) the MMTV retroviral genome consists of approximately 10,000 bp which is very small as compared to the host human genome of 3 billion (3,000,000,000) bp, (ii) the infected cancer cells may be only a small fraction of the cancer specimens, and (iii) the infected cells may contain a low number of the MMTV genome (47). These difficulties can lead to false negative outcomes and probably accounts for the low identification of MMTV by PCR in this current study.

Despite the inconsistent outcomes, results based on PCR are valid for the following reasons: (i) there were variations in approximately 3% of the MMTV *env* gene sequences which is an indication that contamination is unlikely, (ii) the primers used in PCR analyses are based on MMTV gene sequences which are unique to the MMTV genome, (iii) there was no contamination by mouse gene sequences as demonstrated by the absence of MoMt or IAP DNA sequences [these analyses were conducted in the Mount Sinai Medical Center, New York and have been previously published (39)]. In addition, the results based on PCR are consistent with prior studies of MMTV in Australian breast cancers (42, 48). These results are also consistent with US and Italian-based studies (43, 49).

While the antibodies to p14 appear to be specific for the identification of MMTV, it cannot be excluded that some false positive outcomes could occur due to cross-reacting proteins. However, as shown in Figure 7 there was both positive and negative identification of MMTV in benign and breast cancer specimens by immunohistochemistry using p14 antibody, which indicates that p14 antibody is likely to be specific The similar results between the two independent laboratories (Pisa and the University of NSW) is a strong indication that p14-based immunohistochemistry is a more reliable technique than PCR for the identification of MMTV in human breast cancer.

The comparisons of the histologic characteristics of human breast cancers and mouse mammary tumors have obvious subjective elements. Importantly, other cancers such as basal cell carcinomas of the skin have similar characteristics to MMTV positive human breast cancer. For this reason the diagnosis of MMTV involvement in human breast cancer cannot be made on histological characteristics in isolation from additional laboratory evidence. However, the two assessors (James S. Lawson and Wendy K. Glenn) could not distinguish between the MMTV positive human breast cancers and mouse mammary tumors at a microscopic level. At a macroscopic level, human and mouse breast cancers differ.

There are precedents for viral infections leading to cancers with specific histological characteristics. The best known are (i) the association between human papilloma virus infections and koilocytes (cells with characteristic haloes surrounding the nucleus) and (ii) Reed–Sternberg cells (enlarged lymphocytes sometimes with multiple nuclei) in Hodgkin lymphomas. With respect to MMTV and histological characteristics, the similarity between MMTV positive human breast cancers and MMTV positive mouse mammary tumors may hint toward a similar tumor–microenvironment interaction.

The histological characteristics of MMTV-associated human breast cancer are similar to those of neuro-endocrine breast cancers. However, it has been demonstrated that MMTV is not associated with these specific breast cancers (50).

Cancer cells were clearly identified from lymphocytes by their different diameter and appearance and by immunohistochemistry. This confirms that the comparisons between MMTV positive human and mouse mammary tumors are not confused by the presence of lymphocytes.

The observation that MMTV p14 protein was identified by immunohistochemistry in 8 (62%) of the 13 prior benign human breast specimens and that each had some characteristics of hyperplasia and that 7 developed MMTV positive Dunn type B human breast cancers is of considerable interest. This confirms the prior observations (on the same specimens) based on PCR (39). This suggests an initial infection by MMTV in benign breast tissues prior to the development of breast cancer in the same individual patients. In addition, the presence of hyperplasia associated with MMTV infection in benign breast tissues suggests an increase in risk of breast cancer. This is the first association of MMTV with this phenomenon.

Based on the present and previous reports (21, 41), we propose p14 as a potential diagnostic tool, as well as a putative predictor of MMTV-associated breast cancer development. Further studies with larger cohorts of patients in different laboratories will be needed to validate our findings. Taken together with our previous studies on murine MMTV-associated tumors (21, 40, 41, 46), we also propose the use of p14-mediated strategies for preventive and therapeutic implications toward MMTV-associated breast cancers. This may be of special significance in the case of MMTV benign hyperplasia where preventive vaccination might be considered in clinical settings.

### Correlations Between MMTV Identification in Human Breast Cancer, Cancer Grade, and Biomarkers

The absence of any correlations between MMTV positive breast cancer and the biomarkers ER, PR, HER2, and p53 confirms the prior observations by the Pogo group (51). The absence of any correlations between the breast cancer stage and MMTV identification also confirms the prior observations (51).

## Conclusion

We hypothesized that because of the close parallels in the biology of MMTV-associated human breast cancer and mouse mammary tumors, there should be consistent histological patterns in MMTV positive human breast cancers which are similar to MMTV-associated mouse mammary tumors. We have shown that this hypothesis is probably true. We have also confirmed by immunohistochemistry analysis using p14 antibodies that MMTV infections of benign breast tissues occurs before the development of MMTV positive breast cancer. When considered in the context of prior published evidence, the implication of these observations is that MMTV is likely to have a role in human breast cancer.

## Ethics Statement

This project was formally considered and approved by the Human Research Ethics Committee of the University of New South Wales, Sydney, Australia. Reference: HC11421.

## Author Contributions

JL, JH, and CM conceived the study. BY prepared the specimens for analysis. PC, MM, and CM conducted the p14-based immunohistochemistry analyses in Pisa, Italy. CN and WG conducted the p14-based immunohistochemistry analyses in Sydney, NSW, Australia. NW supervised the quality control of analyses and specimen assessments in Sydney. JH and OB developed the p14 antibody, conducted quality controls. JL, CN, and WG conducted the histological assessments. All authors were involved in the writing and final approval of the manuscript.

## Conflict of Interest Statement

No author has any financial, intellectual, or interpersonal conflicts of interest. The reviewer [AA] declared a shared affiliation, with no collaboration, with one of the authors [BY] to the handling Editor.

## Footnotes

^1^By informal convention, the term “mammary tumor,” has been used to describe breast cancer in mice and other rodents.
