# Supplementary material for: Association of Mouse Mammary Tumor Virus With Human Breast Cancer: Histology, Immunohistochemistry and Polymerase Chain Reaction Analyses
Source: Front Oncol. 2018 May 7;8:141. doi: 10.3389/fonc.2018.00141 (PMC5950654; doi:10.3389/fonc.2018.00141)
Supplement: Supplementary file 1 [file Table_1.docx]

**Supplementary Table 1.**

**Correlations (p- value) of MMTV identification by PCR and immunohistochemistry using p14 to breast cancer biomarkers (ER, HER2 , PR, and p53), and histological characteristics (Dunn) of breast cancer.**

|  | **Dunn histology** | **ER** | **HER2** | **PR** | **p53** |
| --- | --- | --- | --- | --- | --- |
| **MMTV p14 by IHC (from UNSW laboratory)** | 0.033  (sig) | 0.395  (not sig) | 0.541  (not sig) | 0.182  (not sig) | 0.100  (not sig) |
| **MMTV p14 by IHC (combined UNSW and Pisa laboratories)** | 0.001  (sig) | 0.177  (not sig) | 0.541  (not sig) | 0.182  (not sig) | 0.100  (not sig) |
| **MMTV identification by PCR** | 0.290  (not sig) | 0.387  (not sig) | 0.215  (not sig) | 0.935  (not sig) | 0.927  (not sig) |
